# Inhibition of p53 prevents diabetic cardiomyopathy by preventing early-stage apoptosis and cell senescence, reduced glycolysis, and impaired angiogenesis

**DOI:** 10.1038/s41419-017-0093-5

**Published:** 2018-01-23

**Authors:** Junlian Gu, Shudong Wang, Hua Guo, Yi Tan, Yaqin Liang, Anyun Feng, Qiuju Liu, Chendil Damodaran, Zhiguo Zhang, Bradley B. Keller, Chi Zhang, Lu Cai

**Affiliations:** 1 Ruian Center of Chinese-American Research Institute for Diabetic Complications, the Third Affiliated Hospital of the Wenzhou Medical University, Ruian, China; 2 Chinese-American Research Institute for Diabetic Complications, the School of Pharmaceutical Sciences of the Wenzhou Medical University, Wenzhou, China; 3 the Department of Pediatrics of the University of Louisville, Pediatrics Research Institute, Louisville, KY 40202 USA; 4 Department of Cardiology, the First Hospital of Jilin University, Changchun, 130021 China; 5 Department of Pediatrics, The First Affiliated Hospital of Wenzhou Medical University, Wenzhou, 325000 China; 6 Department of Hematology, the First Hospital of Jilin University, Changchun, 130021 China; 7 Department of Urology, the University of Louisville, Louisville, KY USA; 8 Kosair Charities Pediatric Heart Research Program, Cardiovascular Innovation Institute, University of Louisville, Louisville, KY 40202 USA

## Abstract

Elevated tumor suppressor p53 expression has been associated with heart diseases, including the diabetic heart. However, its precise role in the pathogenesis of diabetic cardiomyopathy (DCM) remains unclear. We hypothesized that the development of DCM is attributed to up-regulated p53-mediated both early cardiac cell death and persistent cell senescence, glycolytic and angiogenetic dysfunctions. The present study investigated the effect of p53 inhibition with its specific inhibitor pifithrin-α (PFT-α) on the pathogenesis of DCM and its associated mechanisms. Type 1 diabetes was induced with multiple low doses of streptozotocin. Both hyperglycemic and age-matched control mice were treated with and without PFT-α five times a week for 2 months and then sacrificed at 3 and 6 months post-diabetes. Treatment with PFT-α significantly prevented the progression of diabetes-induced cardiac remodeling and dysfunction (i.e., DCM). Mechanistically, the inhibition of p53 prevented the cardiac apoptosis during early-stage diabetes (0.5 month), attenuated diabetes-induced cell senescence (3 and 6 months), and improved both glycolytic and angiogenic defects by increasing hypoxia-induced factor (HIF)-1α protein stability and upregulating HIF-1α transcription of specific target genes at 3 and 6 months after diabetes. Therefore, the targeted inhibition of p53 in diabetic individuals may provide a novel approach for the prevention of DCM.

## Introduction

Diabetes mellitus (DM) is one of the major health threats to modern society^1^. Among all diabetic complications, cardiovascular diseases are the primary cause of mortality in diabetic patients. One of the important early cardiac responses to diabetes may be the apoptotic death^2–4^. We and others have shown that diabetes induces cardiac cell death in the hearts of both type 1 and type 2 diabetes (T1DM and T2DM)^3–7^. The significant increase in cardiomyocyte death is observed at the early stage of T1DM mice induced by streptozotocin (STZ)^4,7^ and cardiac specimens from T2DM patients^8^.

Studies have indicated that p53 and associated apoptosis play important roles in the pathogenesis of mechanical stress and ischemia/reperfusion caused acute cardiac diseases^9,10^. Accordingly, the role of p53 dependent pathways in the pathogenesis of diabetes associated cardiac cell death and diabetic cardiomyopathy (DCM) is of great interest. For instance, a short-term study tried to determine if p53 promotes cardiac dysfunction in diabetes via excessive mitochondrial respiration-mediated oxidative stress and lipid accumulation at the 4th week after T1DM induction by single injection of high-dose STZ^11^. However, this study was unable to definitively answer their questions because: (1) at 4 weeks after STZ treatment, mitochondria damage was most likely due to the direct toxicity of a single high-dose (200 mg/kg) of STZ, rather than diabetes^12,13^; and (2) changes in cardiac function within a short period after STZ treatment are also most likely due to the direct effect of STZ^12^. DCM is a chronic disease, therefore, this short-term study was unable to address whether p53 activation plays an important role in the development of DCM at the later stage of diabetes.

Diabetes impairs glucose uptake and utilization and also decreases adaptive neovascularization (angiogenesis); however, the roles of p53 in the pathogenesis of DCM at later stages of diabetes have never been examined. Recent studies indicated that p53 has broader roles, which include its metabolic regulation in cells under both adaptive physiologic and pathologic disease conditions^14^. One example is that p53 is able to dampen glycolysis possibly by disrupting the balance between glycolysis and oxidative phosphorylation^14^. In addition, p53 is also able to disrupt glucose import^15^ via the pentose phosphate pathway^16^.

In the present study we hypothesized that the activation of p53 may both induce cardiac apoptosis at the early stage and also disrupt glycolysis and angiogenesis throughout the pathogenic process. To test this hypothesis, we utilized a T1DM mouse model with STZ as previously described^17^. These diabetic mice were treated with or without pifithrin-α (PFT-α), a specific p53 inhibitor, for 2 months. Functional and pathological changes in the heart were examined at the early (0.5 month), middle (3 months), and late (6 months) stages of STZ induced diabetes. To mechanistically investigate the preventive effect of PFT-α on the pathogenesis of DCM, in vitro primary cultures of the adult cardiomyocytes were also used with small interfering RNAs (siRNAs) of p53 and murine double minute-2 (MDM2).

## Results

### General features of diabetic mice with and without PFT-α treatment

To test whether inhibition of p53 prevented DCM, multiple low-dose STZ (MLD-STZ)-induced DM mice and age-matched controls were treated with either PFT-α or a vehicle for 2 months. These mice were sacrificed at 0.5, 3, and 6 months after DM onset. Representative images of the general appearance of mice from each group are shown in Supplemental Fig. 1, which showed disorganized fur that lacked luster in MLD-STZ-induced DM mice compared to control mice. PFT-α treatment completely prevented the occurrence of these effects in DM/PFT-α mice, observed at the 6th month of DM. The body weight was reduced in DM group, which was recovered in the DM/PFT-α group by PFT-α treatment at either 3 months or 6 months (Supplemental Fig. 2). Echocardiography examination revealed that DM progressively induced cardiac dysfunction, which were observed at the 3 and 6-month time points (Table 1). These echocardiographic changes were partially but significantly prevented in the DM/PFT-α group by PFT-α treatment.

**Table 1:** Protective effect of PFT-α on diabetes-induced cardiac dysfunction

	Ctrl	PFT-α	DM	DM/PFT-α
2W				
IVS;d	0.62 ± 0.02	0.62 ± 0.03	0.61 ± 0.02	0.62 ± 0.04
LVID;d	3.75 ± 0.03	3.74 ± 0.05	3.78 ± 0.02	3.78 ± 0.04
LVPW;d	0.82 ± 0.03	0.85 ± 0.06	0.86 ± 0.03	0.86 ± 0.03
IVS;s	1.01 ± 0.05	1.05 ± 0.05	1.02 ± 0.03	1.02 ± 0.02
LVID;s	2.13 ± 0.05	2.06 ± 0.12	2.22 ± 0.13	2.14 ± 0.08
LVPW;s	1.28 ± 0.04	1.30 ± 0.07	1.29 ± 0.05	1.31 ± 0.02
LV Vol;d	59.87 ± 1.05	59.62 ± 1.31	61.19 ± 1.01	61.13 ± 0.88
LV Vol;s	14.98 ± 0.92	13.71 ± 1.52	16.61 ± 1.24	15.11 ± 1.47
% EF	75.87 ± 2.32	77.01 ± 2.24	72.85 ± 2.62	75.29 ± 2.21
% FS	43.62 ± 1.19	44.98 ± 2.07	41.23 ± 1.86	38.57 ± 1.51
LV Mass	90.77 ± 2.37	94.40 ± 4.70	96.74 ± 4.95	96.54 ± 4.46
3M				
IVS;d	0.60 ± 0.02	0.61 ± 0.03	0.60 ± 0.02	0.62 ± 0.02
LVID;d	3.70 ± 0.02	3.75 ± 0.02	3.87 ± 0.05*	3.82 ± 0.02*
LVPW;d	0.85 ± 0.03	0.87 ± 0.04	0.80 ± 0.04*	0.86 ± 0.03
IVS;s	1.02 ± 0.02	1.05 ± 0.04	0.97 ± 0.02*	1.03 ± 0.02
LVID;s	2.01 ± 0.07	2.02 ± 0.03	2.65 ± 0.12*	2.32 ± 0.05^†^
LVPW;s	1.27 ± 0.02	1.30 ± 0.02	1.28 ± 0.03	1.31 ± 0.02
LV Vol;d	57.98 ± 0.78	59.87 ± 1.05	65.22 ± 1.10*	62.69 ± 1.3^†^
LV Vol;s	13.13 ± 0.90	13.11 ± 0.45	26.75 ± 1.03*	18.79 ± 0.95^†^
% EF	77.39 ± 1.36	78.10 ± 1.66	60.26 ± 2.43*	70.30 ± 1.83^†^
% FS	45.35 ± 1.28	45.99 ± 1.63	30.76 ± 1.06*	39.04 ± 1.24^†^
LV Mass	89.90 ± 2.22	96.25 ± 2.17	98.01 ± 2.42*	100.41 ± 1.87^*^
6M				
IVS;d	0.62 ± 0.02	0.63 ± 0.03	0.57 ± 0.02*	0.63 ± 0.02^†^
LVID;d	3.77 ± 0.02	3.82 ± 0.06	4.21 ± 0.02*	3.95 ± 0.06^†^
LVPW;d	0.86 ± 0.03	0.86 ± 0.05	0.79 ± 0.03*	0.90 ± 0.02^†^
IVS;s	1.03 ± 0.02	1.04 ± 0.04	0.97 ± 0.01*	1.01 ± 0.06
LVID;s	2.13 ± 0.03	2.15 ± 0.10	3.14 ± 0.10*	2.43 ± 0.05^†^
LVPW;s	1.35 ± 0.03	1.37 ± 0.02	1.25 ± 0.02*	1.31 ± 0.02^†^
LV Vol;d	60.66 ± 0.87	63.33 ± 2.23	79.05 ± 0.99*	68.03 ± 2.3^†^
LV Vol;s	14.87 ± 0.62	15.28 ± 1.91	39.10 ± 3.24*	20.74 ± 1.0^†^
% EF	75.47 ± 1.32	75.93 ± 2.20	50.58 ± 3.49*	69.48 ± 1.83^†^
% FS	43.55 ± 1.22	44.04 ± 1.95	25.49 ± 2.13*	38.57 ± 1.51^†^
LV Mass	95.52 ± 1.92	102.06 ± 2.36	105.64 ± 2.84*	108.57 ± 2.17^*^

Notes: *IVS* interventricular septum, *LVID* *d* left ventricular internal diastolic diameter, *LVID* *s* left ventricular internal systolic diameter, *LVPW* left ventricular posterior wall, *EF* ejection fraction, *FS* fractional shortening, *LV* *vol* *s* left ventricular end systolic volume, *LV vol d* *left ventricular end diastolic volume*, *LV mass* left ventricular mass. Data are presented as the mean ± SD (*n* = 6)

*, *p* < 0.05 vs. Ctrl group; †, *p* < 0.05 vs. DM group

### Effect of PFT-α on DM-induced cardiac remodeling, inflammation, and oxidative stress

**Fig. 1:**
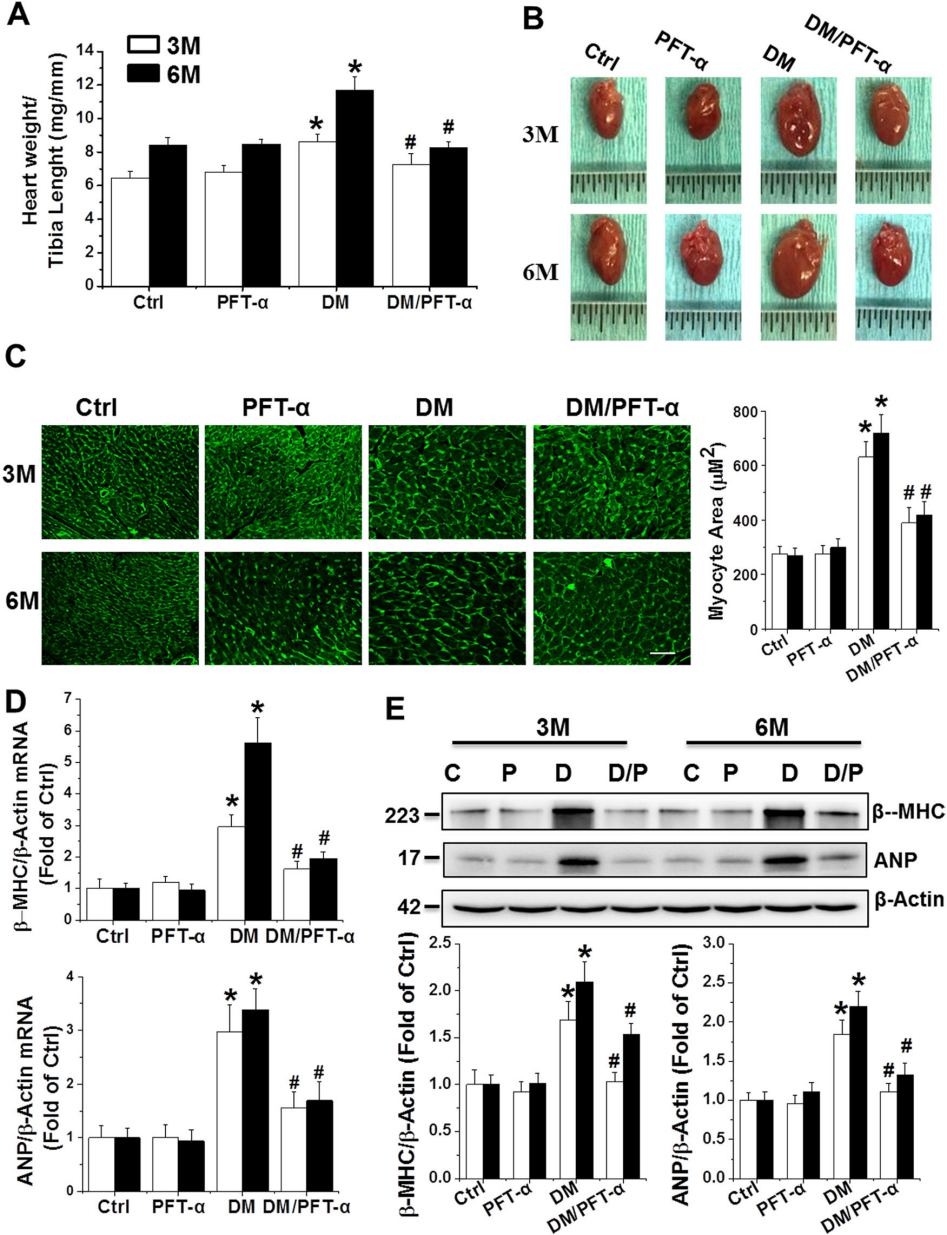
Effect of PFT-α treatment on diabetes-induced cardiac hypertrophy **a** Heart weight to tibia length ratio. **b** Heart size. **c** Cardiac tissue FITC-conjugated WGA staining and quantification of myocyte cross-sectional areas (Scale bar = 25 µm). **d, e** qRT-PCR and western blot analysis of hypertrophic markers ANP and β-MHC mRNA (**d**) and protein (**e**) expressions. C: Ctrl; P: PFT-α; D: DM; D/P: DM/PFT-α

The ratio of heart weight to tibia length significantly increased in the DM group at the 3 and 6-month time points, which is suggestive of cardiac hypertrophy, an effect that was completely prevented by PFT-α treatment (Fig. 1a). Diabetes-induced cardiac hypertrophy was further confirmed by the observation of enlarged hearts in the experimental mice (Fig. 1b). Hypertrophic cardiomyocytes were evident by semi-quantitative analysis of the myocyte area with wheat germ agglutinin (WGA) staining (Fig. 1c). Morphological hypertrophy was further supported by the upregulation of the molecular hypertrophy markers atrial natriuretic peptide (ANP) and β-myosin heavy chain (β-MHC) at the mRNA (Fig. 1d) and protein levels (Fig. 1e) in the DM mice, but not in the PFT-α/DM mice (Fig. 1a–e).

**Fig. 2:**
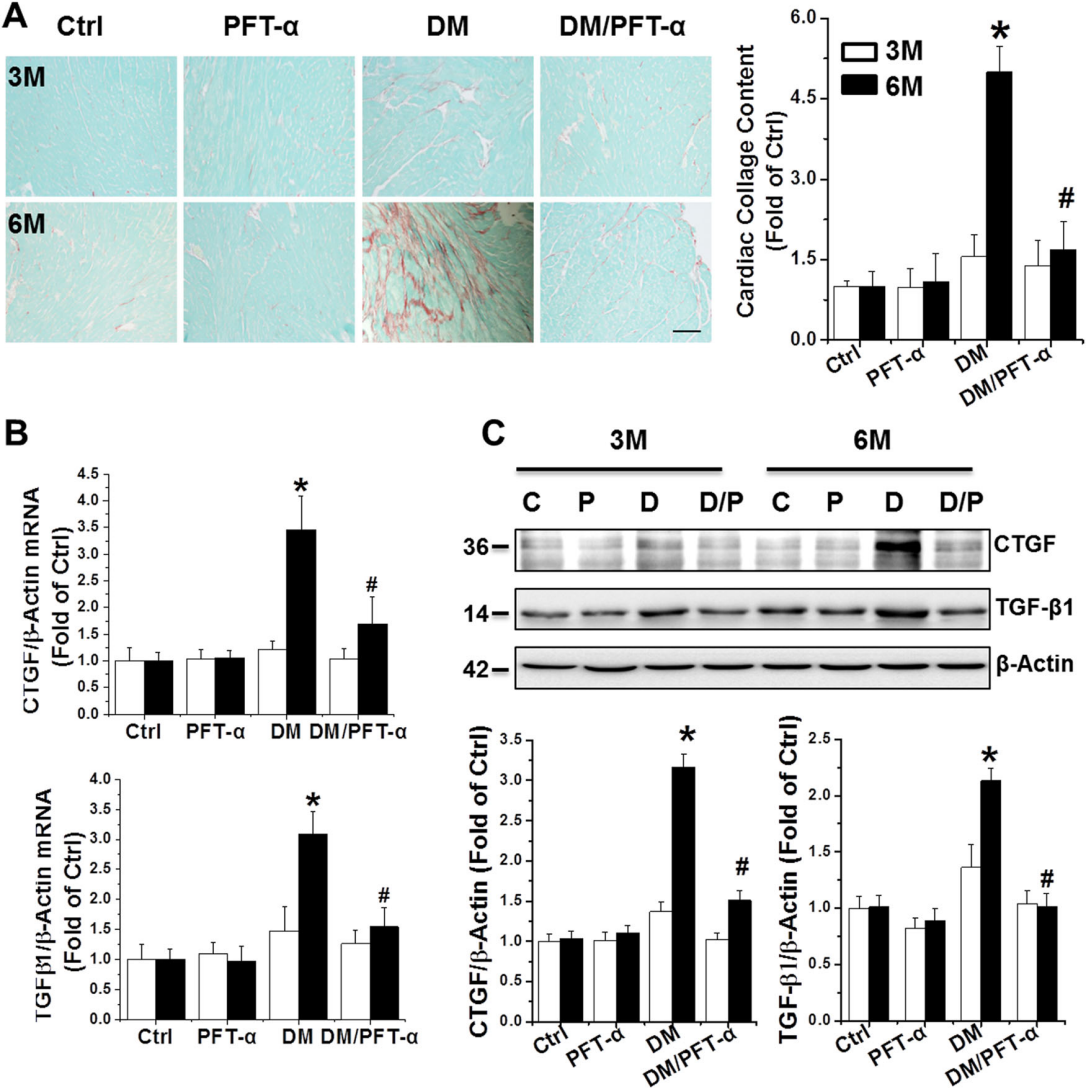
Effect of PFT-α treatment on diabetes-induced cardiac fibrosis **a** Cardiac fibrosis, determined by Sirius red staining of collagen accumulation (collagen is red, Scale bar = 25 µm) and quantitative analysis. **b, c** mRNA (**b**) and protein (**c**) expression of CTGF and TGF-β1 examined by qRT-PCR and western blot analysis. Data presented as mean ± SD (*n* = 6). *P < 0.05 vs. Ctrl; #, *P* < 0.05 vs. DM

Sirius red staining revealed significant collagen accumulation in DM mice at 6 months (Fig. 2a), suggesting the induction of cardiac fibrosis. Cardiac fibrosis was confirmed by qRT-PCR and western blot of the pro-fibrotic mediators, connective tissue growth factor (CTGF) and transforming growth factor β1 (TGF-β1) (Figs. 2b, c). None of these fibrotic indices were significantly increased in DM/PFT-α mice (Fig. 2a–c).

**Fig. 3:**
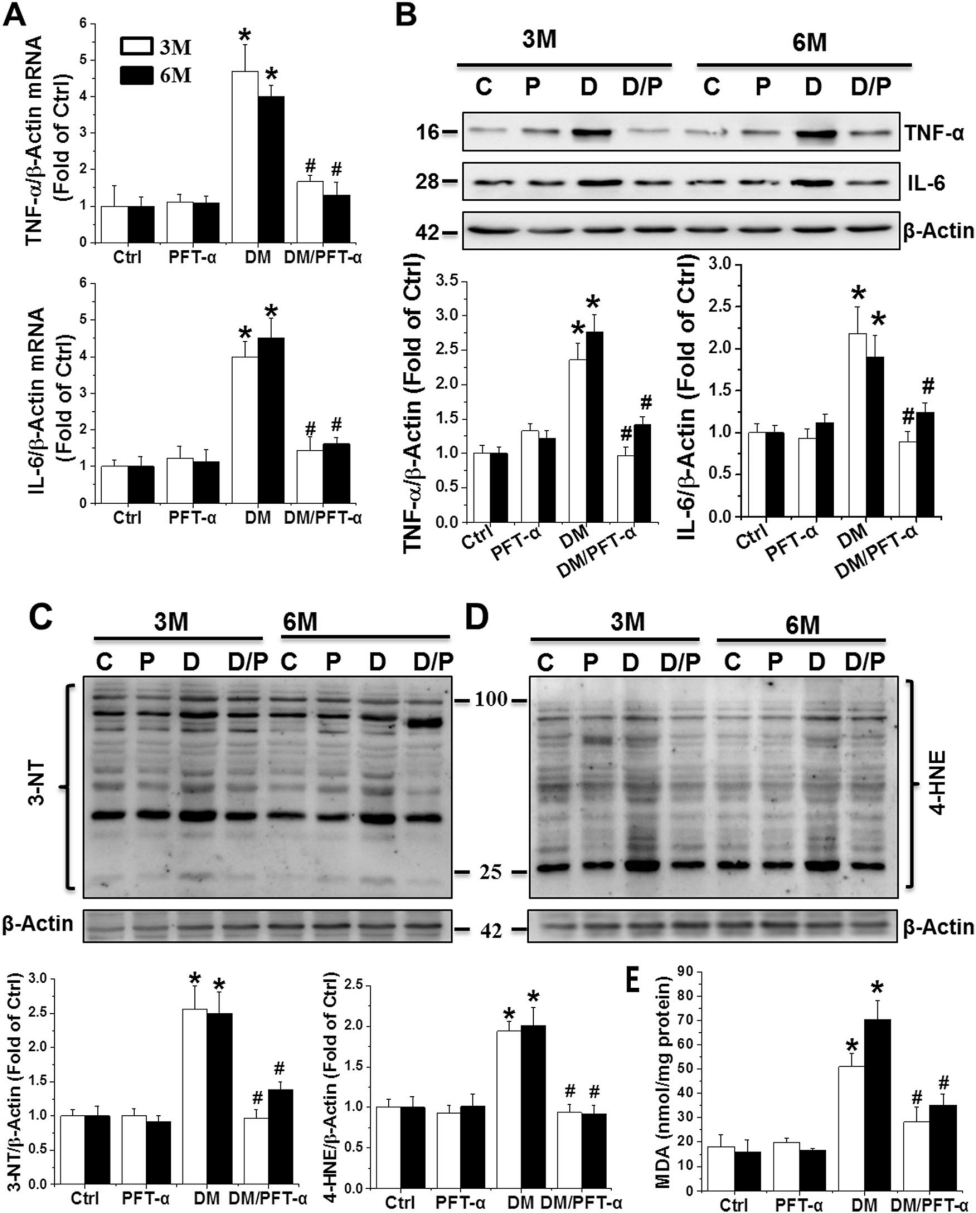
Effect of PFT-α treatment on diabetes-induced cardiac inflammation and oxidative stress **a, b** Cardiac inflammation measured by mRNA and protein expression of TNF-α and IL-6 by qRT-PCR (A) and western blot analysis (**b**). (**c**–**e**) Oxidative stress detected by 3-NT (**c**) and 4-HNE (**d**) protein levels as well as MDA accumulation (**e**). Data presented as mean ± SD (*n* = 6). *P < 0.05 vs. Ctrl; #*P* < 0.05 vs. DM

We next examined cardiac expression of inflammatory cytokines tumor necrosis factor alpha (TNF-α) and interleukin-6 (IL-6) by qRT-PCR (Fig. 3a) and western blot (Fig. 3b). Significantly increased TNF-α and IL-6 mRNA expression and protein levels were noted in the DM group, which was completely prevented by PFT-α treatment.

**Fig. 4:**
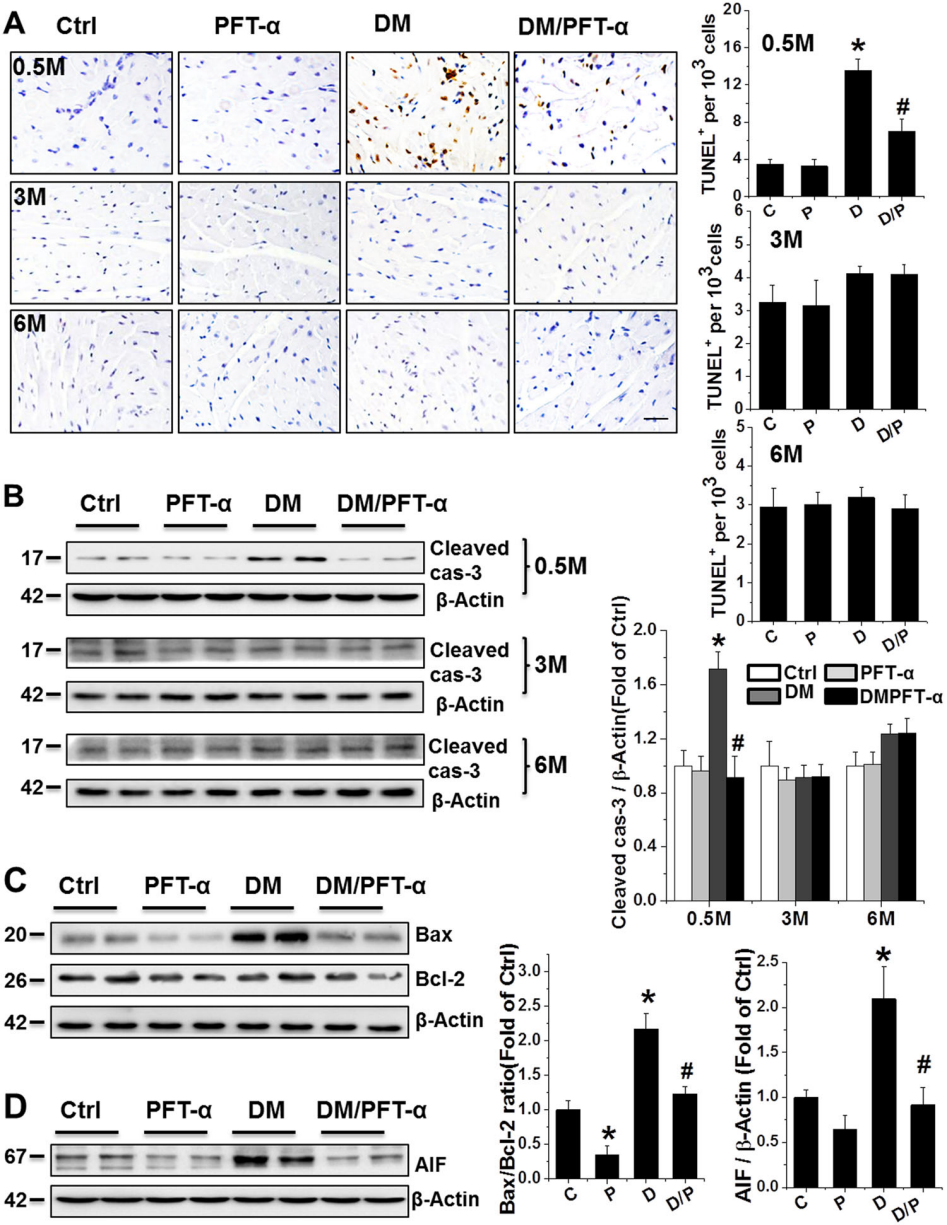

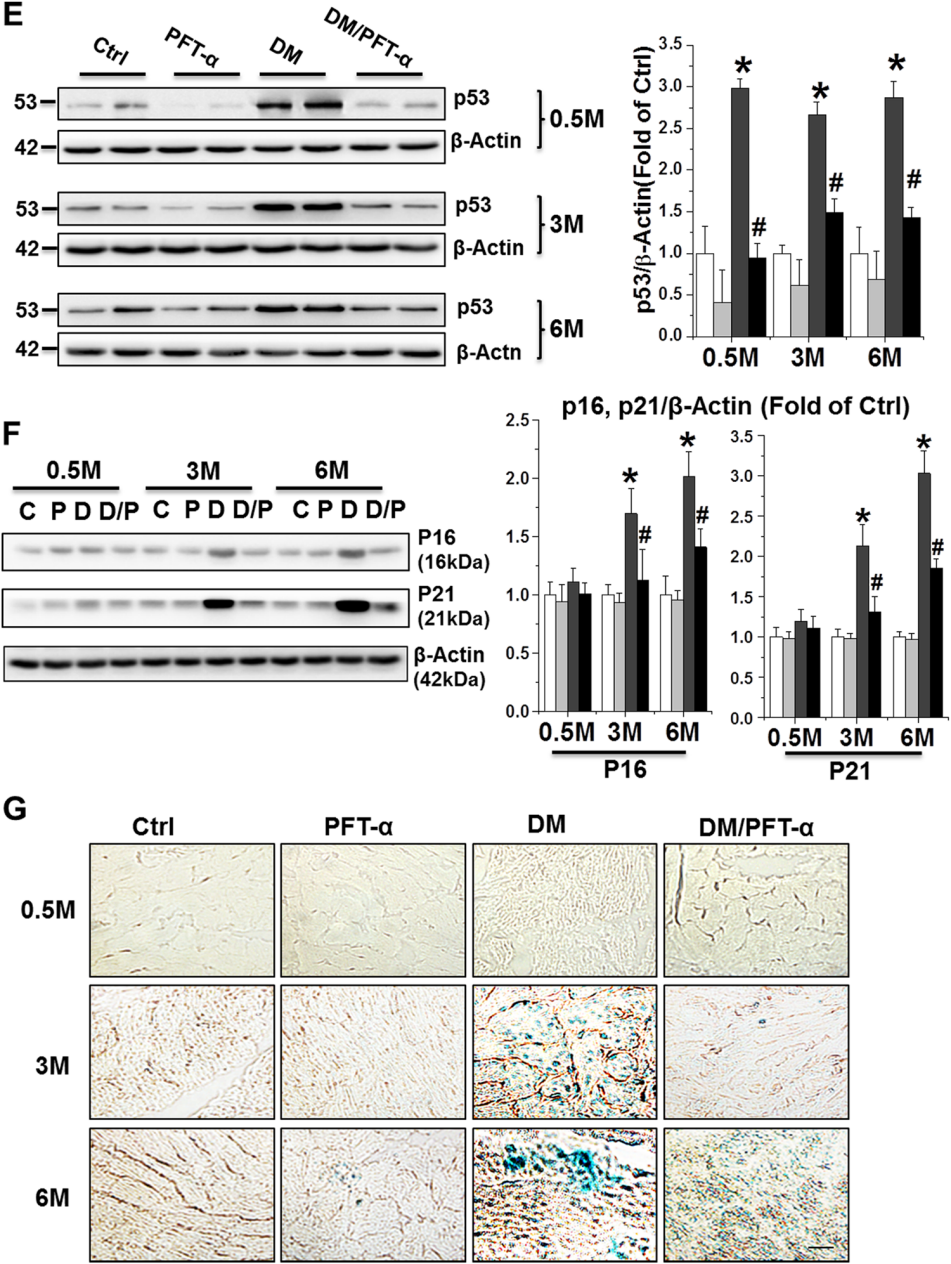
Effect of PFT-α treatment on mitochondrial apoptotic cell death and cell senescence **a** Representative images of TUNEL fluorescent staining (Scale bar = 25 µm) at 0.5-, 3-, and 6-month after diabetes onset. **b** Protein expression of cleaved caspase-3 at 0.5, 3, and 6-month after diabetes onset examined using western blot assays. **c, d** Protein expression of Bax/Bcl-2 ratio (**c**), and AIF (**d**) at 0.5 month after diabetes onset examined using western blot assays. **e, f** Protein expression of p53, p16INK4A (p16) and p21WAF1/CIP (p21) examined by western blot analysis of heart tissues at 0.5, 3, and 6-month time points. **g** SA-β-gal activity stained at 0.5, 3, and 6-month after diabetes onset. Scale bar = 25 µm. Data presented as mean ± SD (*n* = 6). *P < 0.05 vs. Ctrl; #*P* < 0.05 vs. DM

Oxidative stress has been suggested to play a key role in DM-induced cardiac pathogenesis^18^ and was measured by protein nitration and peroxidation with western blot of 3-nitrotyrosine (3-NT) (Figs. 3c) and 4-hydroxy-2-nonenal (4-HNE) (Fig. 3d). Lipid peroxidation was detected using the malondialdehyde (MDA) assay (Fig. 3e). Both oxidative stress indices were significantly increased in the DM mice, but not in the PFT-α/DM mice.

### Effect of PFT-α on diabetes-induced cell death and cell senescence

Increased numbers of TUNEL-positive cells were observed in the hearts of DM mice only at 0.5 month after DM onset compared to control mice, and were almost completely absent after PFT-α treatment (Fig. 4a). Mitochondria-dependent apoptotic cell death is prevalent in the T1DM mouse heart^4^, highlighting the role of mitochondrial cell death pathways in T1DM-induced apoptosis. Thus, p53-mediated-mitochondrial cell death pathway was examined in our PFT-α treatment paradigm. Western blot revealed a significant increase in the Bax to Bcl-2 ratio and apoptosis-inducing factor (AIF) expression, which are both an index of mitochondrial cell death pathway^19,20^, along with increased level of cleaved caspase-3 in the DM group, but not in the PFT-α/DM mice (Figs. 4b–d).

It should be noted that although there was no significant increase in caspase-3 cleavage and TUNEL-positive cells at late stages of DM (3 and 6 months, Figs. 4a,b), consistent with our and other previous studies^4,5,7^, Western blot (Fig. 4e) revealed a significant increase in p53 protein expression both at the early stage (0.5 month) and at other time points (3 and 6 months) in DM groups. In addition, a persistent inhibition of p53 expression was observed in the DM/PFT-α group from 0.5 to 6 months after DM onset. These results suggest that the prevention of DCM by inhibition of p53 may be due to both the suppression of early apoptotic cell death and the prevention of later pathogenic effects, such as increased cell senescence. Therefore, we investigated the effect of PFT-α on cell senescence at the 0.5, 3, and 6-month time points. Diabetes increased cell senescence, as reflected by increased expression of senescence markers (p16INK4A and p21WAF1/CIP) (Fig. 4f) and senescence-associated β-galactosidase (SA-β-gal) activity (Fig. 4g) at the 3 and 6 months DM time points, and these cell senescence indices were significantly attenuated by PFT-α.

### Effect of PFT-α treatment on glucose metabolism and angiogenesis

**Fig. 5:**
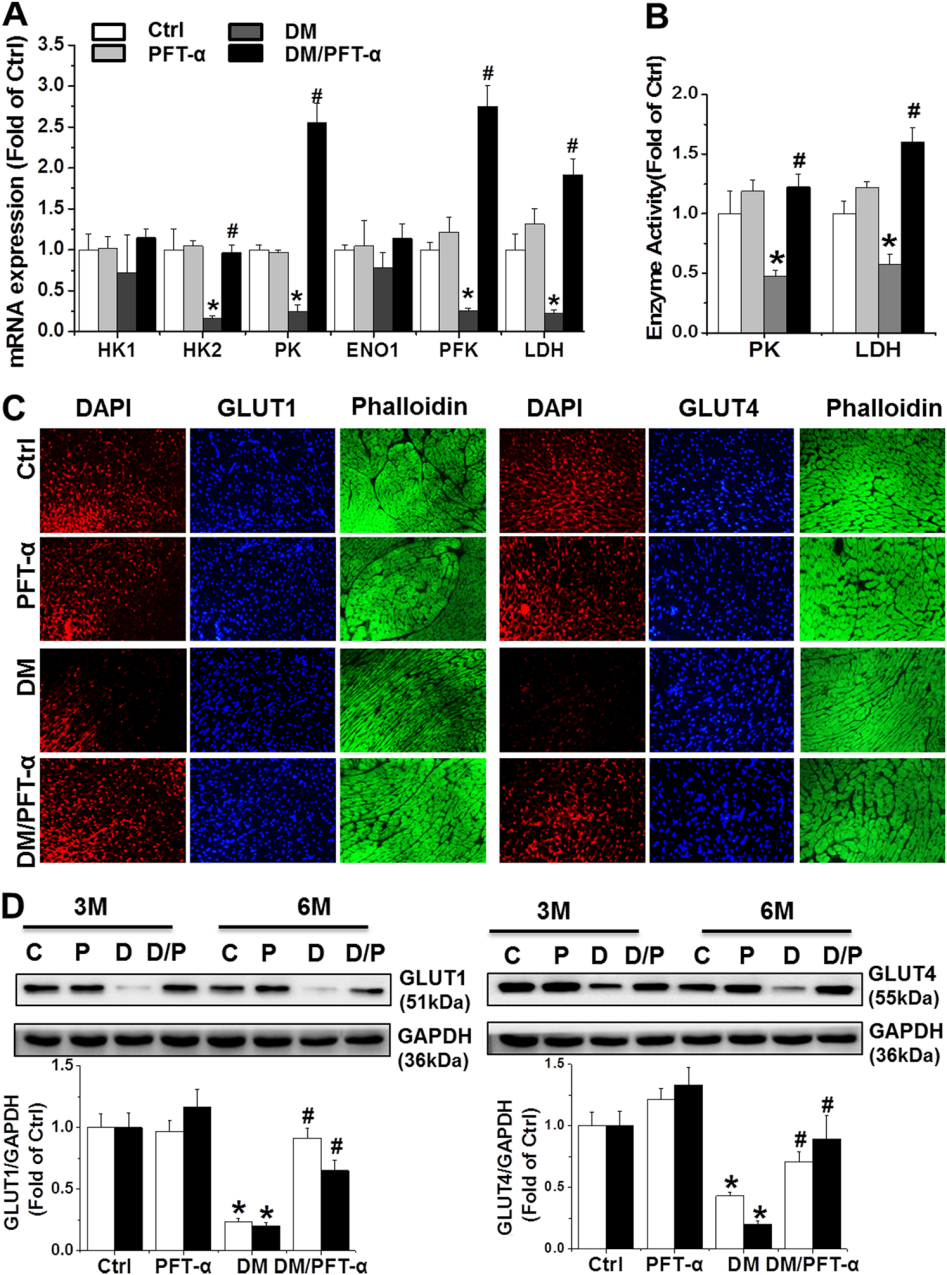

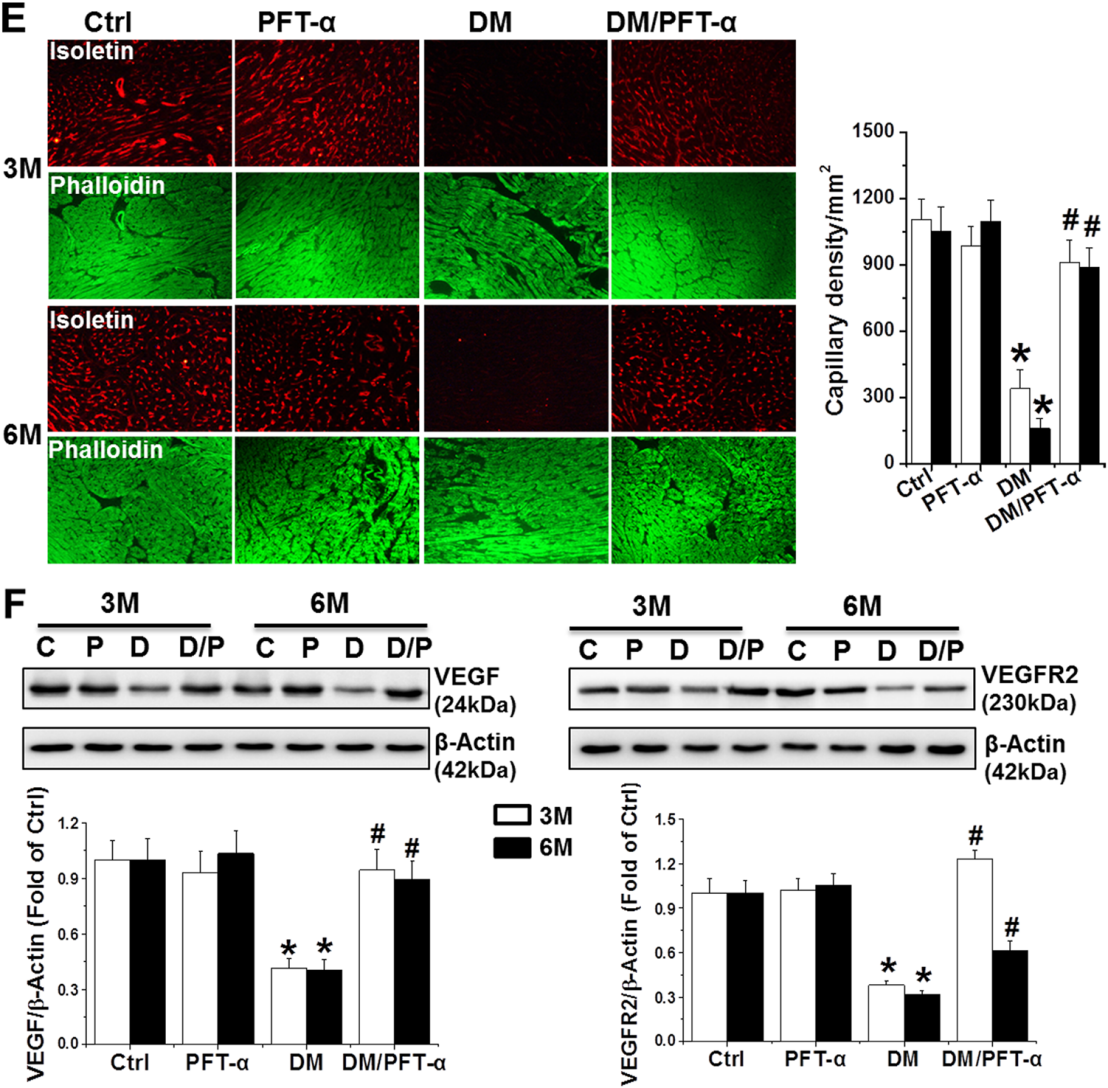
Effect of PFT-α treatment on glucose metabolism and angiogenesis **a** qRT-PCR analysis of glycolysis related enzymes mRNA expression as indicated at 6 months after diabetes onset. **b** PK and LDH activities detected at 6 months after diabetes onset. (**c, d**) Expression of GLUT1 and GLUT4 detected by immunofluorescence, by which GLUT1 or GLUT4 were stained as red, DAPI as blue for nuclei, and phalloidin as green for actin fibers (**c**) (Scale bar = 25μm) at the 6-month time points and western blot analysis (**d**) at the 3 and 6-month time points. **e** Capillary density in the 3 and 6-month diabetic and control heart tissues assessed by isolectin GS-IB4 staining (Red) with phalloidin for actin fibers (Green). Scale bar = 25 μm. **f** Expression of VEGF and its receptors VEGFR2 detected by western blot at the 3 and 6-month time points. Data presented as mean ± SD (*n* = 6). *P < 0.05 vs. Ctrl; #*P* < 0.05 vs. DM

To further investigate the other pathological effects of p53 activation beyond cell death and senescence, we investigated the effect of PFT-α on the mRNA levels of key glycolytic enzymes including hexokinase 1 (HK-1), HK-2, pyruvate kinase (PK), enolase 1(ENO1), phosphofructokinase (PFK), and lactate dehydrogenase (LDH), as well as PK and LDH activities, as previously described^21^. The results showed that PFT-α either preserved or significantly increased the mRNA levels of HK-2, PK, PFK, and LDH as well as the activity of PK and LDH in the DM/PFT-α mice at the 3 and 6-month time points relative to that in the DM mice (Supplemental Figs. 3a, b; Figs. 5a, b).

Reduced glucose uptake in the diabetic heart is associated with decreased activity of glucose transporters such as glucose transporter (GLUT) 1, and 4^22,23^. Thus, we determined the protein expression of GLUT-1 and GLUT-4 by immunofluorescence (Fig. 5c) and western blot analysis (Fig. 5d). DM mice at the 3 and 6-month time points showed a decrease in GLUT-1 and GLUT4 protein expression compared to control mice and PFT-α treatment markedly normalized DM-reduced GLUT-1 and GLUT4 protein expression. In parallel to GLUT-1 and GLUT-4 expression, the high glucose (HG)-treated primary cardiomyocytes showed a reduction in glucose uptake compared to that in the control group, an effect that was largely prevented by PFT-α or p53-siRNA treatment (Supplemental Fig. 4a–c).

The second major function of HIF-1α is to stimulate cardiac angiogenesis in response to hypoxic conditions such as chronic DM. Insufficiency in blood vessel formation in the diabetic heart is characterized by a significant reduction in the expression of vascular endothelial growth factor (VEGF) and its receptor, VEGFR2^23^. We showed that PFT-α treatment did not induce changes in capillary density in the non-DM myocardium but significantly prevented DM-induced reduction at the 3 and 6-month time points (Fig. 5e). In addition, consistent with the observed capillary density, a significant reduction in VEGF and VEGR2 expression was observed in the DM group, but not in the DM/PFT-α group (Fig. 5f).

### p53 interacts with HIF-1α, which facilitates MDM2-dependent ubiquitination and consequent proteasomal degradation in late-stage DM

**Fig. 6:**
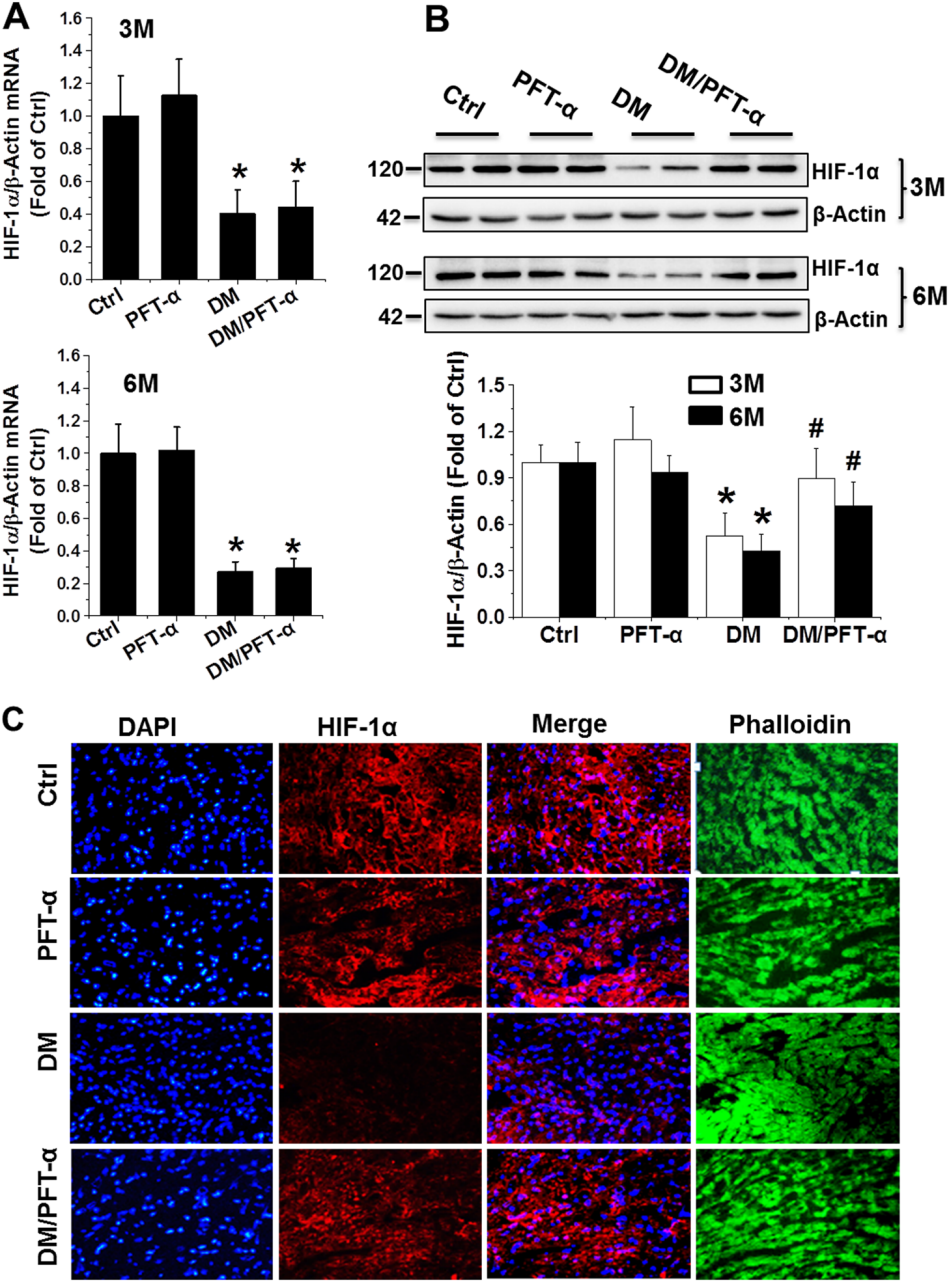

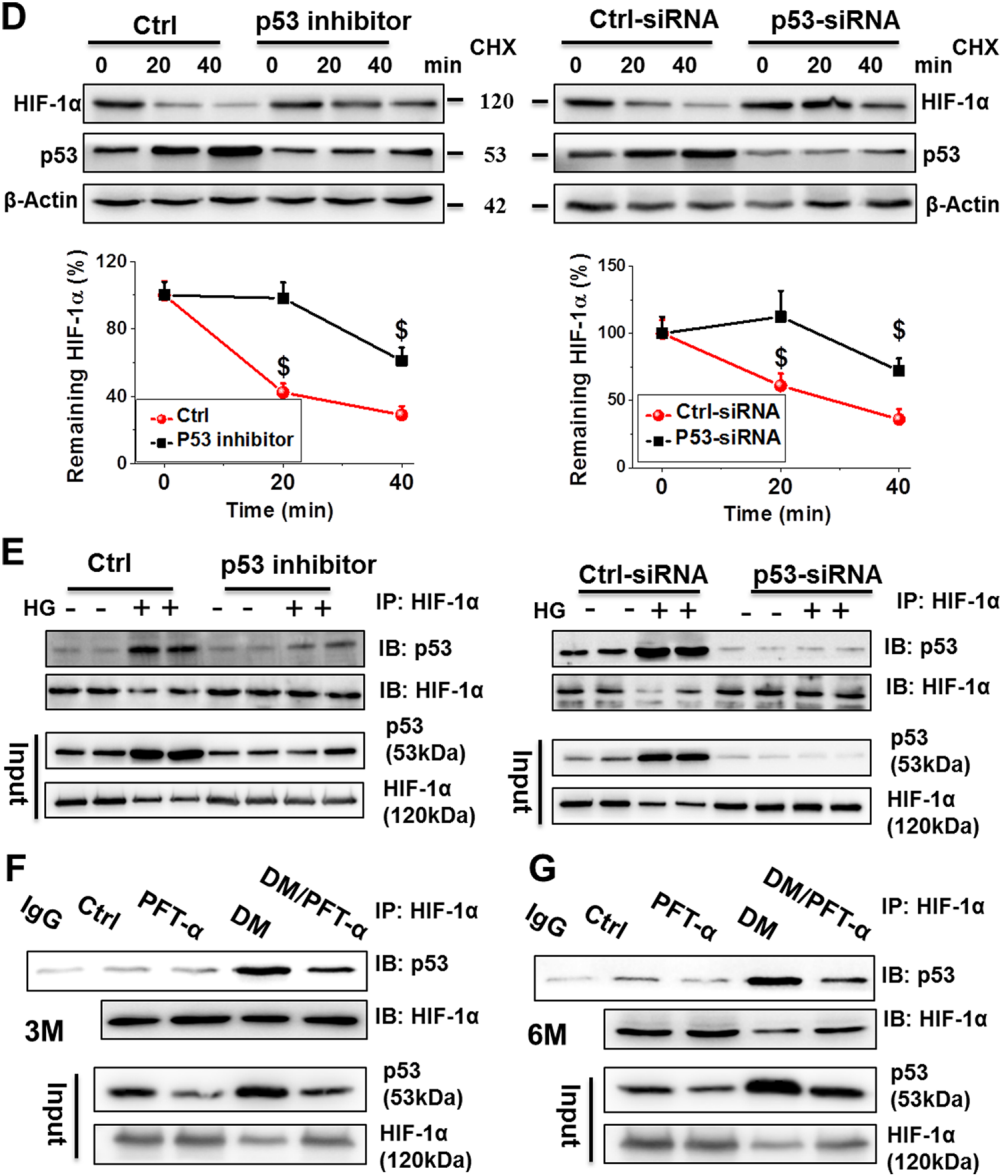
p53 controls HIF-1α protein expression at 3 and 6 months after diabetes onset **a, b** The mRNA and protein expression of HIF-1α examined by qRT-PCR (**a**) and western blot (**b**) analysis of heart tissues at 3 and 6 months after diabetes onset. **c** Immunofluorescence staining using anti-HIF-1α antibody (red), DAPI for nuclei (blue), and phalloidin for actin fibers (green) of heart tissues at 6 months after diabetes onset (Scale bar = 25 µm). **d** A time course of 100 μM cycloheximide (CHX) treatment used to analyze the HIF-1α half-life in primary cardiomyocytes with or without PFT-α or p53-siRNA. β-actin used as loading control. (E-G) Protein-protein interaction between p53 and HIF-1α after PFT-α or p53-siRNA treatment in both primary cardiomyocytes **e** and heart tissues at 3 **f** and 6 **g** months after diabetes onset demonstrated by immunoprecipitation in the presence of the proteasomal inhibitor MG132. Data expressed as mean ± SD of three independent experiments. *P < 0.05 vs. Ctrl; #*P* < 0.05 vs. DM. $*P* < 0.05 vs. 0 min

Defective glycolysis and angiogenesis in the hearts of patients with DM and experimental DM animals have been reported^23^. To explore the molecular mechanism of p53 inhibition on glycolysis and angiogenesis, we examined the mRNA and protein expression of HIF-1α, a key transcription factor for the hypoxic induction of glucose utilization and angiogenetic factors, in the hearts of DM mice with and without PFT-α treatment. The results showed that HIF-1α mRNA levels significantly decreased in the DM group compared to that in the control mice. But PFT-α treatment did not increase its mRNA levels in DM/PFT-α mice (Fig. 6a). In contrast to HIF-1α mRNA levels, HIF-1α protein levels were almost completely normalized by PFT-α treatment in DM mice at the 3 and 6-month time points (Fig. 6b). HIF-1α immunofluorescence was further confirmed, in which the DM-downregulated cardiac HIF-1α protein expression in DM mice was prevented by PFT-α treatment (Fig. 6c; Supplemental Fig. 5).

To investigate a potential mechanism by which p53 inhibition resulted in the preservation of HIF-1α protein expression following PFT-α treatment the half-life of HIF-1α was analyzed in primary cardiomyocytes under HG conditions. In control-transfected or treated cells, the HIF-1α protein underwent decay at a half-life of 20–40 min, whereas this was extended beyond 40 min in p53 siRNA-transfected or PFT-α-treated cells (Fig. 6d). These findings indicated that p53 negatively controls the levels of the HIF-1α protein. To investigate the molecular mechanism by which p53 regulates HIF-1α protein levels, we next determined whether, similar to p73^24^, a direct interaction of p53 with HIF-1α is involved. Immunoprecipitation using the protein lysate of primary cardiomyocytes revealed the coexistence of p53 and HIF-1α in the same molecular complex (Fig. 6e). Similarly, protein interactions were also confirmed by immunoprecipitation of cardiac tissues (Figs. 6f, g).

**Fig. 7:**
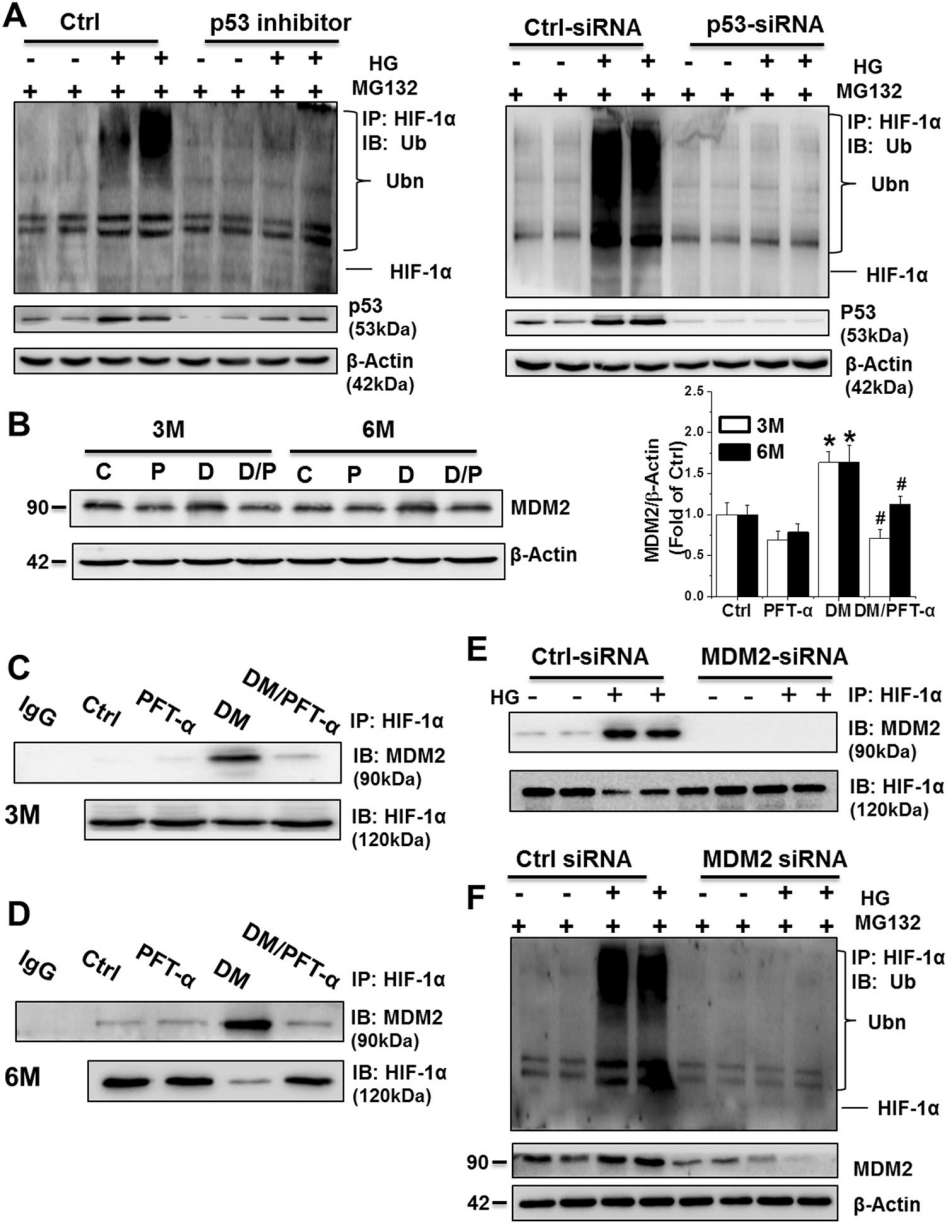
p53 facilitates HIF-1α ubiquitination and consequent proteasomal degradation in an MDM2-dependent manner **a** HIF-1α polyubiquitination examined by immunoprecipitation and western blot of primary cardiomyocytes in the presence of MG132 with or without PFT-α or p53-siRNA treatment. **b** The MDM2 protein expression detected by western blot analysis. **c, d** The protein-protein interaction between MDM2 and HIF-1α detected at 3 (**c**) and 6 (**d**) months after diabetes onset by immunoprecipitation in vivo. **e** The protein-protein interaction between MDM2 and HIF-1α detected by immunoprecipitation in primary cardiomyocytes after transfection with Ctrl-siRNA or MDM2-siRNA. **f** HIF-1α polyubiquitination examined by immunoprecipitation and western blot in primary cardiomyocytes after transfection with Ctrl-siRNA or MDM2-siRNA. Data expressed as mean ± SD of three independent experiments. *P < 0.05 vs. Ctrl; #*P* < 0.05 vs. DM

To determine whether the observed increase in HIF-1α ubiquitination was a consequence of the p53-HIF1-α interaction, we then analyzed ubiquitin-HIF-1α levels with or without PFT-α or p53 siRNA treatment. In the presence of the 20 s proteasome inhibitor, MG132, HG strongly increased the expression of the ubiquitinated forms of HIF-1α, and the ubiquitinated level of HIF-1α was significantly reduced by PFT-α or p53 siRNA treatment under HG conditions (Fig. 7a). Thus, p53 promoted HIF-1α ubiquitination, leading to proteasomal degradation, through a protein-protein interaction with HIF-1α.

Ubiquitin conjugation to proteins destined for proteasomal degradation involves a complicated process that includes an E1 Ub-activating enzyme, E2 Ub-conjugating enzyme, and E3 Ub-protein ligase. Multiple E3 Ub-ligase enzymes are responsible for the selection of proteins destined for the proteasome. MDM2 as one of E3 Ub-protein ligases is able to bind to p73 at the N-terminal region and inhibits tumor angiogenesis by promoting HIF-1α degradation^24^. We hypothesized that the p53-HIF-1α interaction also facilitates MDM2-dependent HIF-1α ubiquitination, with p53 playing the role of a scaffold protein. First, we detected the protein expression of MDM2 among groups at various time points. Western blot and immunofluorescence analysis indicated the upregulation of MDM2 in the DM groups, but not in the DM/PFT-α groups, at the 3 and 6-month time points relative to that in the control mice (Fig. 7b; Supplemental Fig. 6). At the 3 and 6-month time points, anti-HIF-1α immunoprecipitation with MDM2 from the DM group, but not the DM/PFT-α group, displayed significantly higher MDM2 levels in the complex compared to that in the other groups (Figs. 7c, d), thereby suggesting that the interaction of MDM2 with HIF-1α under DM conditions is p53-dependent. A similar experiment involving selective knockdown of endogenous MDM2 in primary cardiomyocytes resulted in the disruption of the HIF-1α-MDM2 interaction and a reduction in the levels of ubiquitinated HIF-1α when MDM2 was depleted (Figs. 7e, f).

## Discussion

This is the first study, to our knowledge, to investigate whether targeting p53 using PFT-α prevents cardiac tissue damage in a long-term T1DM model (until 6 months after diabetes). One major finding is that the prevention by p53 inhibition of DM-induced cardiac remodeling and dysfunction are associated with both attenuation of the early-stage apoptosis and prevention of the persistent cell senescence and glycolytic and angiogenetic dysfunction.

We previously reported that DM-induced cardiac apoptosis occurs at an early stage (7–21 days) in mitochondria-dependent manner^4^. In line with these studies, the present study shows that DM induces a significant increase in the number of apoptotic cells, mirrored by increased TUNEL-positive cells, expression of cleaved caspase-3, ratio of Bax to Bcl2 expression, and AIF expression exclusively at the 0.5 month time-point (Fig. 4). PFT-α treatment resulted in a significant reduction of apoptotic effect at 0.5 month after DM. It is noticed that, at 3 and 6 months after DM, although there is still a higher p53 expression in the DM group compared to control, the rate of DM-induced apoptosis was significantly low, as indicated by the levels of cleaved caspase-3 expression and the results of the TUNEL assay (Fig. 4).

To date, no report has explained why a low apoptotic rate is observed in late-stage DM despite significant cardiac dysfunction. Our explanation is that cardiac cells may respond via apoptotic death at early stage but respond via cell senescence at the relatively late stages since significant increases in senescent markers (Fig. 4). Adult cardiomyocytes are terminally differentiated, and thus cell loss can be detrimental to cardiac function. When cardiomyocytes are lost, collagen is deposited to replace apoptotic cardiomyocytes^25^, which lead to cardiac fibrosis and dysfunction. Although senescent cells were not removed like apoptotic cells, these cells do not have normal function and even generate inflammatory cytokines, leading to microenvironmental inflammatory responses and oxidative stress (Fig. 3)^26^; To support this notion, a recent study reports that enhancing clearance of senescent cells could reverse effects of chemotoxicity and aging in aged mice by preventing p53 nuclear transcription^27^. In our model we also confirmed inhibition of p53 have significantly reduced senescent cells from 3 to 6 months after the onset of DM.

Late-stage DM hearts are also characterized by downregulation of HIF-1α (Fig. 6). HIF-1α is a master transcription factor that confers cardioprotection by triggering the activation of specific downstream genes^21^. Stabilization of HIF-1α by inhibition of prolyl hydroxylase or by treatment with CoC_12_ reduced cardiac infarct size and cardiac dysfunction after global ischemia/reperfusion^28^. However, whether inhibition of p53 can preserve HIF-1α activity in the DM heart has not yet been investigated. An earlier study showed that p53 causes cardiac hypertrophy during pressure overload by inhibition of HIF-1α^29^. Another study demonstrated that p53 promotes the ubiquitin-mediated degradation of HIF-1α via recruitment of an E3 ubiquitin–protein ligase (MDM2) and subsequently inhibits the angiogenic switch during tumorigenesis^30^. Here we demonstrated for the first time in the mouse model of type 1 diabetes conditions that p53 also directly increases the expression of MDM2 and recruits MDM2 to HIF-1α, thereby facilitating its ubiquitin-dependent proteasomal degradation and inhibiting its transactivation properties under DM conditions. Of note, after p53 inhibitor treatment, HIF-1α protein stability and activity was significantly improved (Figs. 5, 6, and 7).

Except for inducing apoptotic cell death, p53 also has multiple functions via upregulating HIF-1α transcription of its specific downstream genes including the glucose transporters, GULT1 and GULT4. Previous reports have shown that the expression of GLUT-1 and GLUT-4 are reduced in the DM heart^22,23^, which in turn leads to reduction in cardiac glucose influx and uptake in patients with DM. Similar to the results of previous studies, STZ-induced DM significantly reduced both GLUT-1 and GLUT-4 protein expression and glucose uptake in the heart (Figs. 5c, d and Supplemental Fig. 4), which can be reversed by p53 inhibition. In addition to glucose transporters, key glycolytic enzymes such as PK and LDH play important roles in cellular glucose metabolism, thereby maintaining the downhill glucose concentration gradient that is necessary for glucose transport into cells^21^. The reduction in activity of these glycolytic enzymes is believed to be a mechanism in DCM, and restoration of cardiac glycolysis improves DM heart function^23^. The present study determined that PFT-α treatment increases the activity of key glycolytic enzymes such as PK and LDH in the DM heart via the upregulation of HIF-1α (Figs. 5a, b and Supplemental Figs. 3a, b).

Another important gene that is regulated by HIF-1α is VEGF. VEGF is a key regulator of angiogenesis in the myocardium. Impaired myocardial VEGF expression along with inadequate angiogenesis has been shown to be a major cellular and metabolic response in diabetic cardiomyopathy^31^. In the present study, we found that the inhibition of p53 results in the restoration of DM-associated reduced VEGF and reduced VEGF receptor levels, which subsequently leads to the improvement of capillary density in the DM heart (Figs. 5e, f).

One innovative finding of the present study is that treatment of DM mice with PFT-α for the first 2 months elicited a persistent cardioprotective effect on DM, which was observed not only during the treatment with PFT-α at 0.5 month, but also at 1 and 4 months after discontinuation of PFT-α treatment (i.e., the 3th and 6th month of DM). Although the underlying mechanism for the persistence of cardiac protection by short inhibition of p53 remains unclear, we assume that it may be related to the epigenetic regulation of p53 expression based on the following facts. PFT-α was reported able to reverse cancer-downregulated the activity of DNA methyltransferases (DNMTs) DNMT1 and DNMT3a in HCT-116 colon cancer cells, suggesting the possible for p53 directly or indirectly to increase DNMTs activity^32^. Reportedly PFT-α blocks interaction of p53 with transcriptional cofactor p300 and downregulates histone acetylation after DNA damage^33^.

In summary, we demonstrated here for the first time that inhibition of p53 prevents the functional and pathological abnormalities of the heart in a T1DM mouse model, through multiple mechanisms, as illustrated in Supplemental Fig. 7. During the early stage of DM (0.5 month), inhibition of p53 reduces cardiac mitochondrial cell death, whereas during late-stage DM (3–6 months), inhibition of p53 prevents DM-induced cardiac senescence and impairment of glucose metabolism and angiogenesis. Regarding the improvement of glucose metabolism and angiogenesis by inhibition of p53 is most likely mediated by increasing HIF-1α protein stability and its transcription of specific target genes. However, its direct clinical importance remains elusive and thus further exploration is warranted.

## Materials and methods

### Animals and treatment

FVB male mice were purchased from The Jackson Laboratory. All experimental procedures were approved by the Institutional Animal Care and Use Committee of the University of Louisville. FVB mice were injected intraperitoneally with MLD-STZ (50 mg/kg body weight) [Sigma-Aldrich, St. Louis, MO, USA] daily for five consecutive days. We used MLD-STZ-induced T1DM mouse model because although our previous studies showed that a single high dose of STZ (150–210 mg/kg) induces T1DM, these diabetic mice have high mortality due to the systemic toxicity of high-dose STZ^4,34^, whereas MLD-STZ induces T1DM in mice without mortality^17,35^. Mice with hyperglycemia (blood glucose levels ≥ 250 mg/dL) were defined as diabetic. These mice were divided into four groups (*n* = 6): non-diabetic (Ctrl), PFT-α-treated non-diabetic (PFT-α), diabetic mice (DM), and DM with PFT-α (DM/PFT-α). PFT-α (Sigma-Aldrich) was intraperitoneally administered at 1.1 mg/kg five times a week for 2 months based on published studies^36^, and then mice were kept for additional 4 months without PFT-α treatment. Mice were sacrificed at 0.5, 3, and 6 months post-diabetes for various measurements. 6 months after diabetes as the longest time-point was selected since our previous studies have extensively confirmed the well-developed DCM at this time-point^17,18,34,35^. Usage of the selective p53 inhibitor, PFT-α, rather than p53 knockout mice, in the present study because of (1) the similar effects of PFT-α and p53 deficient mice on p53 function^36–38^, (2) inhibition of p53 after diabetes onset; (3) also inhibition of p53 only for short period (2 months) to investigate whether temporal inhibition can provide a persistent effect after stopping use of inhibitor. In addition, compared to genetic approaches, the pharmacological inhibition of p53 in diabetic individuals may provide a novel approach for the prevention of DCM in the future.

### Echocardiography

Transthoracic echocardiography was performed using a high-resolution imaging system for small animals (Vevo 770, Visual Sonics) as previously described^34,35,39^.

### Western blot and quantitative real-time PCR (qRT-PCR)

Western blot and qRT-PCR were performed according to our previous studies^4,17,39^. Antibodies and primers are listed in Supplemental Table 1 and 2.

### Histological staining

Cardiac fibrosis was detected with a mixture of 0.1% Sirius red F3BA and 0.25% Fast Green FCF and quantified using Pro Plus 6.0 software^39^. Cardiac cell death was detected by Terminal deoxynucleotidyl transferase-mediated dUTP nick end labeling (TUNEL) staining and presented as the number of TUNEL-positive cells per 10^3^ cells^40^. For myocyte cross-sectional histological analysis, sections were stained with Alexa Fluor® 488 conjugated WGA and calculated using ImageJ software^39^. SA-β-gal activity was measured with a SA-β-gal staining kit (Cell Signaling, Danvers, MA, USA).

### Biochemical measurement of lipid peroxides

To detect lipid peroxide accumulation in the heart, the tissue protein was determined by measuring thiobarbituric acid reactivity, reflected by the content of MDA formed during acid hydrolysis of the lipid peroxide compound, as previously described^39^.

### Immunofluorescence

Immunofluorescence was performed as previously described^17,39^. Antibodies are listed in Supplemental Table 1. Images were captured with Nikon microscope (Nikon, Melville, NY, USA) and quantified using the NIH ImageJ 1.43 software.

### Primary cardiomyocyte isolation and treatment

Adult mouse primary cardiomyocytes were isolated and cultured as previously described^35,41^. Primary cardiomyocytes were treated for 48 h with HG (25 mM glucose) and PFT-α (30 µM) or a specific siRNAs against p53 and MDM2 (Invitrogen, Carlsbad, CA, USA). The siRNA transfections in adult mouse cardiomyocytes were performed using Lipofectamine® 3000 (Invitrogen) following the manufacturer’s instructions as previously described^35^.

### Immunoprecipitation

Immunoprecipitation was performed with heart tissues (30 mg from each mouse) or cell lysates (500 µg) lysed in immunoprecipitation buffer using a Pierce Co-Immunoprecipitation kit (Pierce Biotechnology Ltd., Rockford, IL, USA) as previously described^42–44^. Immunocomplexes were determined using specific antibodies as indicated.

### Statistical analysis

Data from all replicates were presented as the mean ± SD (*n* ≥ 6 in each group). Comparisons among various groups were performed by two-way ANOVA, followed by post-hoc pairwise repetitive comparisons using Tukey’s test with Origin 9.0 Lab data analysis and graphing software. Statistical significance was considered as *P* < 0.05.

## Electronic supplementary material

Supplementary data
