# Supplementary material for: Inhibition of p53 prevents diabetic cardiomyopathy by preventing early-stage apoptosis and cell senescence, reduced glycolysis, and impaired angiogenesis
Source: Cell Death Dis. 2018 Jan 23;9(2):82. doi: 10.1038/s41419-017-0093-5 (PMC5833384; doi:10.1038/s41419-017-0093-5)
Supplement: Supplementary file 1 — Supplementary data [file 41419_2017_93_MOESM1_ESM.docx]

**Inhibition of p53 prevents diabetic cardiomyopathy by preventing early-stage apoptosis and cell senescence, reduced glycolysis, and impaired angiogenesis**

Junlian Gu ^1,2,3^, Shudong Wang ^4^, Hua Guo ^3^, Yi Tan ^1,2,3^, Yaqin Liang ^5^, Anyun Feng ^1^, Qiuju Liu ^6^, Chendil Damodaran ^7^, Zhiguo Zhang ^4^, Bradley B. Keller ^3,8^, Chi Zhang ^1,2,^*, Lu Cai ^1,2,3^

**Contents**

1. **Supplemental Tables**
2. **Supplemental Figures**

**1. Supplemental Tables**

**1.1. Supplemental Table 1: List of antibody for western blot and immunofluorescence**

| **Number** | **Name** | **Company** | **Dilution** |
| --- | --- | --- | --- |
| **For western blot** | | |  |
| **1** | Interleukin-6 (IL-6) | Santa Cruz Biotechnology | 1;500 |
| **2** | Connective tissue growth factor (CTGF) | Santa Cruz Biotechnology | 1;1000 |
| **3** | Glyceraldehyde 3-phosphate dehydrogenase (GAPDH) | Santa Cruz Biotechnology | 1;3000 |
| **4** | β-actin | Santa Cruz Biotechnology | 1;3000 |
| **5** | Atrial natriuretic peptide (ANP) | Santa Cruz Biotechnology | 1;500 |
| **6** | Glucose transporter (GLUT) 1, and 4 | Santa Cruz Biotechnology | 1;500 |
| **7** | p53 | Cell Signaling | 1;500 |
| **8** | Cleaved caspase-3 | Cell Signaling | 1;1000 |
| **9** | Bax | Cell Signaling | 1;1000 |
| **10** | Bcl-2 | Cell Signaling | 1;1000 |
| **11** | Apoptosis-inducing factor (AIF) | Cell Signaling | 1;1000 |
| **13** | MDM2 | Cell Signaling | 1;500 |
| **14** | Ubiquitin | Abcam | 1;1000 |
| **15** | Tumor necrosis factor alpha (TNF-α) | Abcam | 1;1000 |
| **16** | Vascular endothelial growth factor (VEGF) | Abcam | 1;500 |
| **17** | β-myosin heavy chain (β-MHC) | Abcam | 1;500 |
| **18** | VEGF receptor 2 (VEGFR2) | Abcam | 1;500 |
| **19** | Hypoxia-induced factor (HIF)-1α | Novus Biologicals | 1;500 |
| **20** | Transforming growth factor β1 (TGF-β1) | Abcam | 1;1000 |
| **21** | 3-nitrotyrosine (3-NT) | Millipore | 1;2000 |
| **21** | 4-hydroxy-2-nonenal (4-HNE) | Alpha Diagnostic International | 1;2000 |
| **22** | HRP-conjugated IgG | Abcam | 1;3000 |
| **For immunofluorescence** | | |  |
| **23** | Hypoxia-induced factor (HIF)-1α | Novus Biologicals | 1;100 |
| **24** | Isolectin GS-IB4 | Invitrogen | 1;100 |
| **25** | Phalloidin | Thermal Fisher | 1;50 |
| **26** | Glucose transporter (GLUT) 1, and 4 | Santa Cruz Biotechnology | 1;50 |
| **27** | Cy3-conjugated IgG | Abcam | 1;200 |
| **28** | FITC-conjugated IgG | Abcam | 1;100 |
| **29** | DAPI | Sigma-Aldrich | 1;3000 |

**1.2. Supplemental Table 2: List of primer for quantitative real-time PCR (qRT-PCR)**

| **Number** | **Name** | **Company** | **Cat No** |
| --- | --- | --- | --- |
| **1** | β-Actin | Applied Biosystems | Mm02619580 |
| **2** | IL-6 | Applied Biosystems | Mm00446190 |
| **3** | TNF-α | Applied Biosystems | Mm00443258 |
| **4** | ANP | Applied Biosystems | Mm01255474 |
| **5** | β-MHC | Applied Biosystems | Mm01319006 |
| **6** | Hexokinase 1 (HK1) | Applied Biosystems | Mm00439344 |
| **7** | Hexokinase 2 (HK2) | Applied Biosystems | Mm00443385 |
| **8** | Phosphofructokinase (PFK) | Applied Biosystems | Mm01309576 |
| **9** | Enolase 1 (ENO1) | Applied Biosystems | Mm01619597 |
| **10** | Pyruvate kinase (PK) | Applied Biosystems | Mm00834102 |
| **11** | Lactate dehydrogenase (LDH) | Applied Biosystems | Mm00496648 |
| **12** | CTGF | Applied Biosystems | Mm01192933 |
| **13** | TGF-β1 | Applied Biosystems | Mm01178820 |
| **14** | HIF-1α | Applied Biosystems | Mm00468869 |

1. **Supplemental Figures**


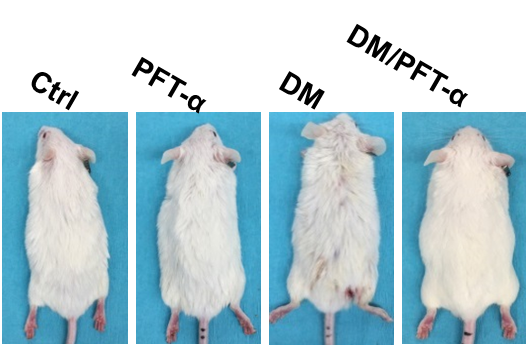


Supplemental Fig. 1. Effect of PFT-α treatment on the general features in STZ-induced diabetic mice


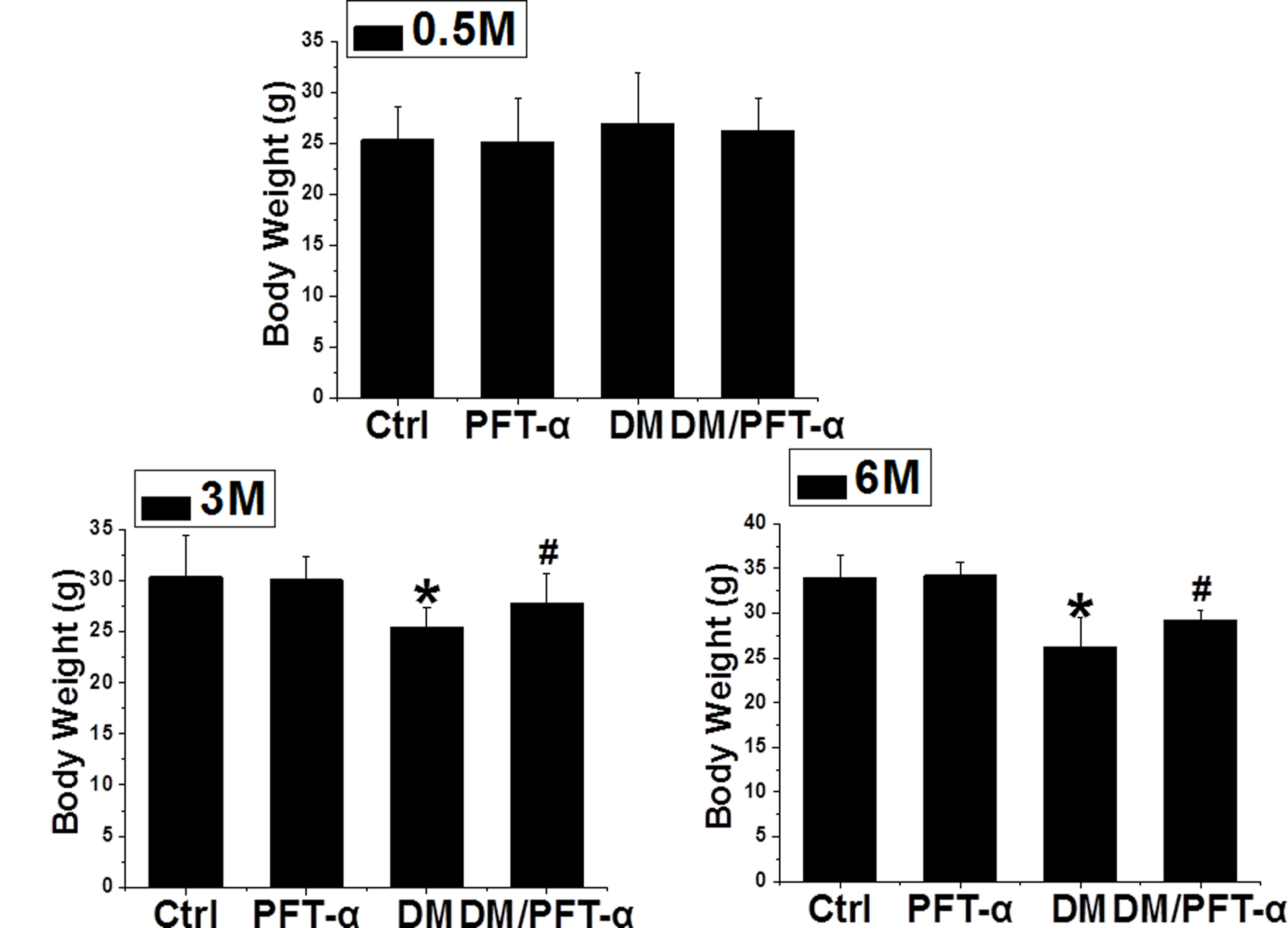


Supplemental Fig. 2. Effect of PFT-α treatment on body weight at 0.5-, 3- and 6-month after diabetes onset. Data expressed as mean ± SD (n=6). *, P < 0.05 vs. Ctrl; #, P < 0.05 vs. DM.


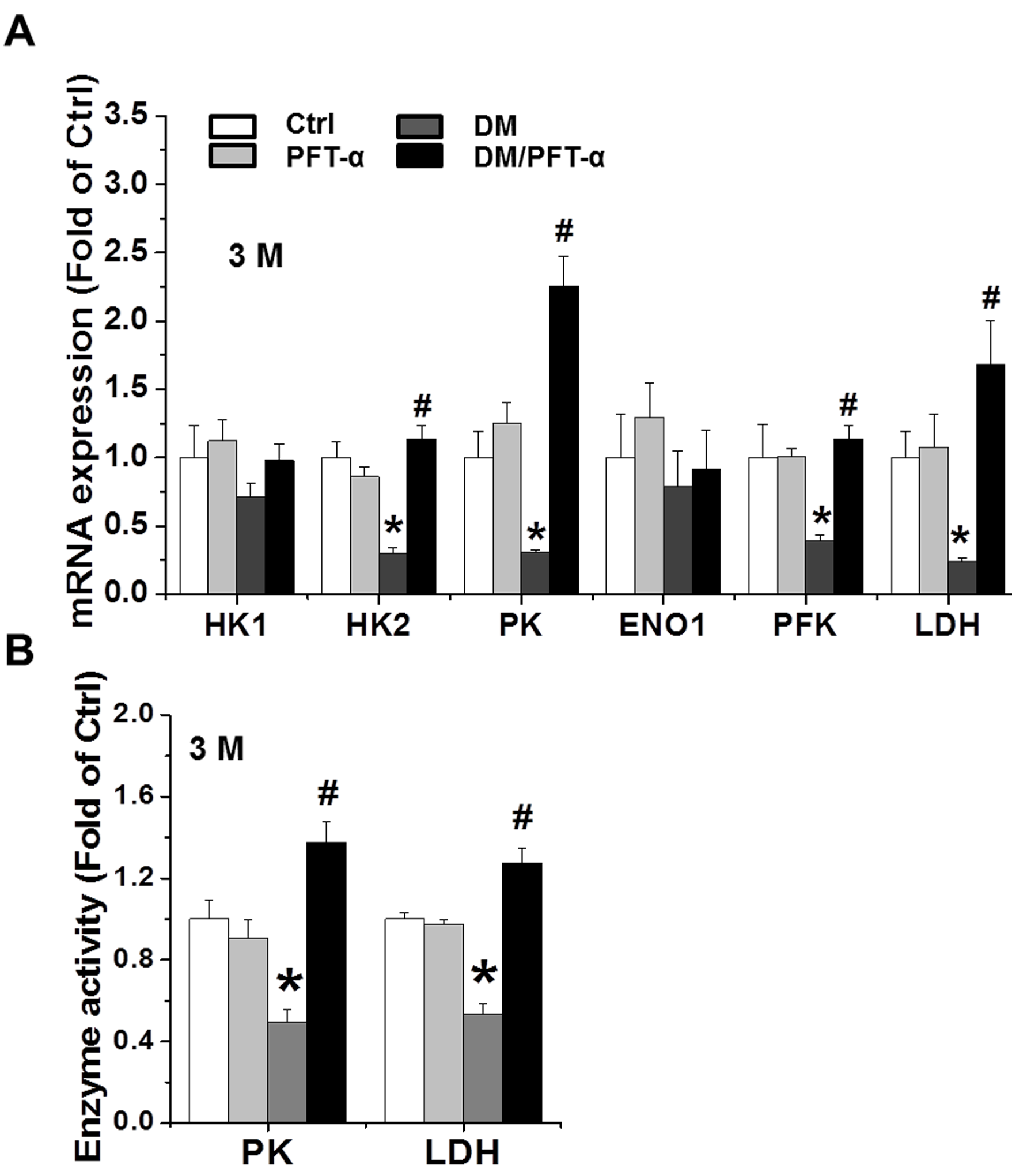


Supplemental Fig. 3. Effect of PFT-α treatment on glucose metabolism at the 3-month time point. (A) qRT-PCR analysis of mRNA expression of glycolysis-related enzymes. (B) PK and LDH activities detected at 3 months after diabetes onset. Data expressed as mean ± SD (n=6). *, P < 0.05 *vs*. Ctrl; #, P < 0.05 *vs.* DM.


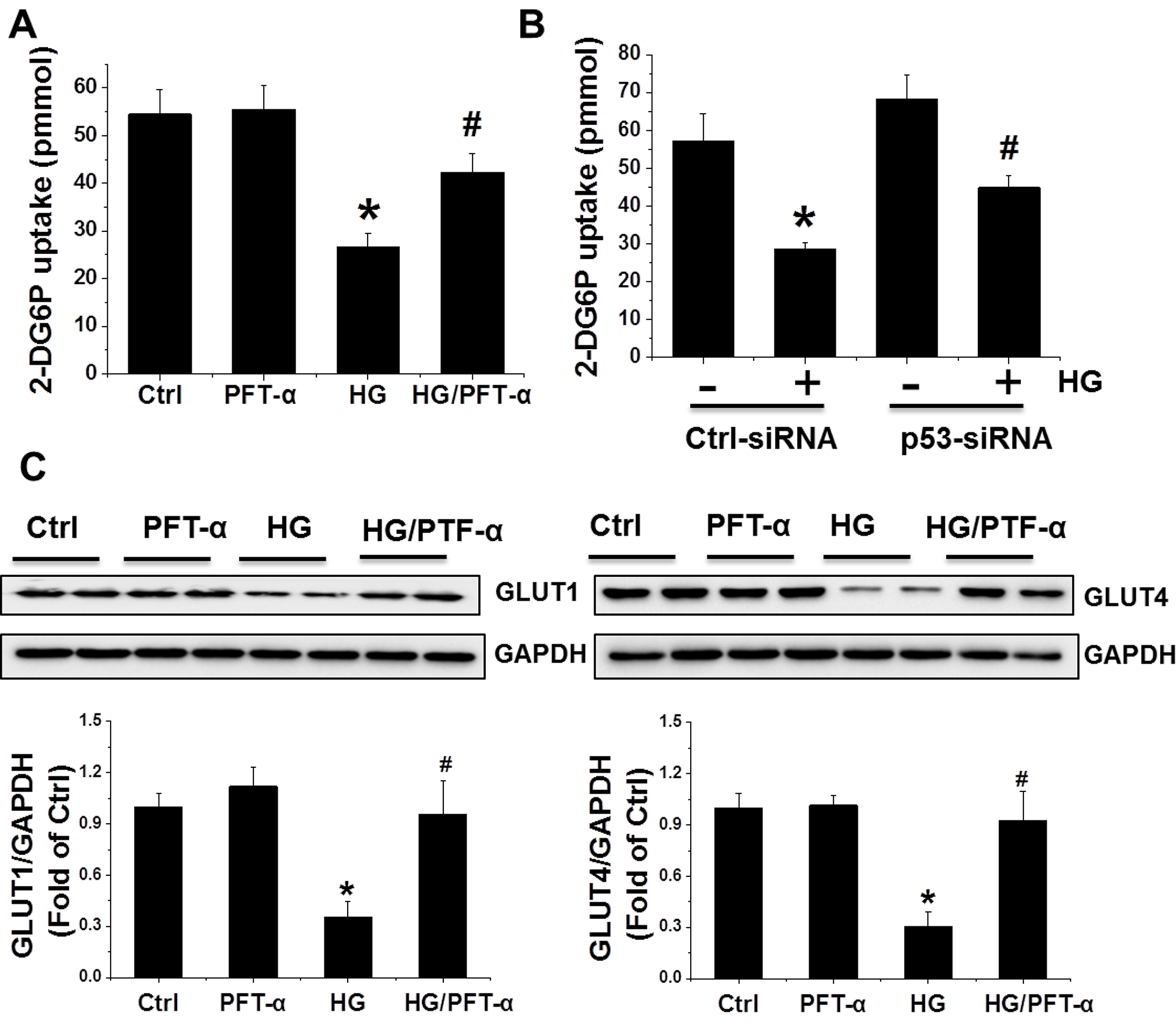


Supplemental Fig. 4. Effect of PFT-α treatment on glucose uptake in primary cardiomyocytes. (A-B) Glucose uptake detected in primary cardiomyocytes after different treatment . (C) Protein expression of GLUT1 and GLUT4 detected by western blotting. Data expressed as mean ± SD of three independent experiments. *, P < 0.05 vs. Ctrl; #, P < 0.05 vs. HG. The molecular weight of GLUT1 (51 kD) and GLUT4 (55KD) are similar to that of actin (42KD), and thus GAPDH (36 kD) was selected as loading control.


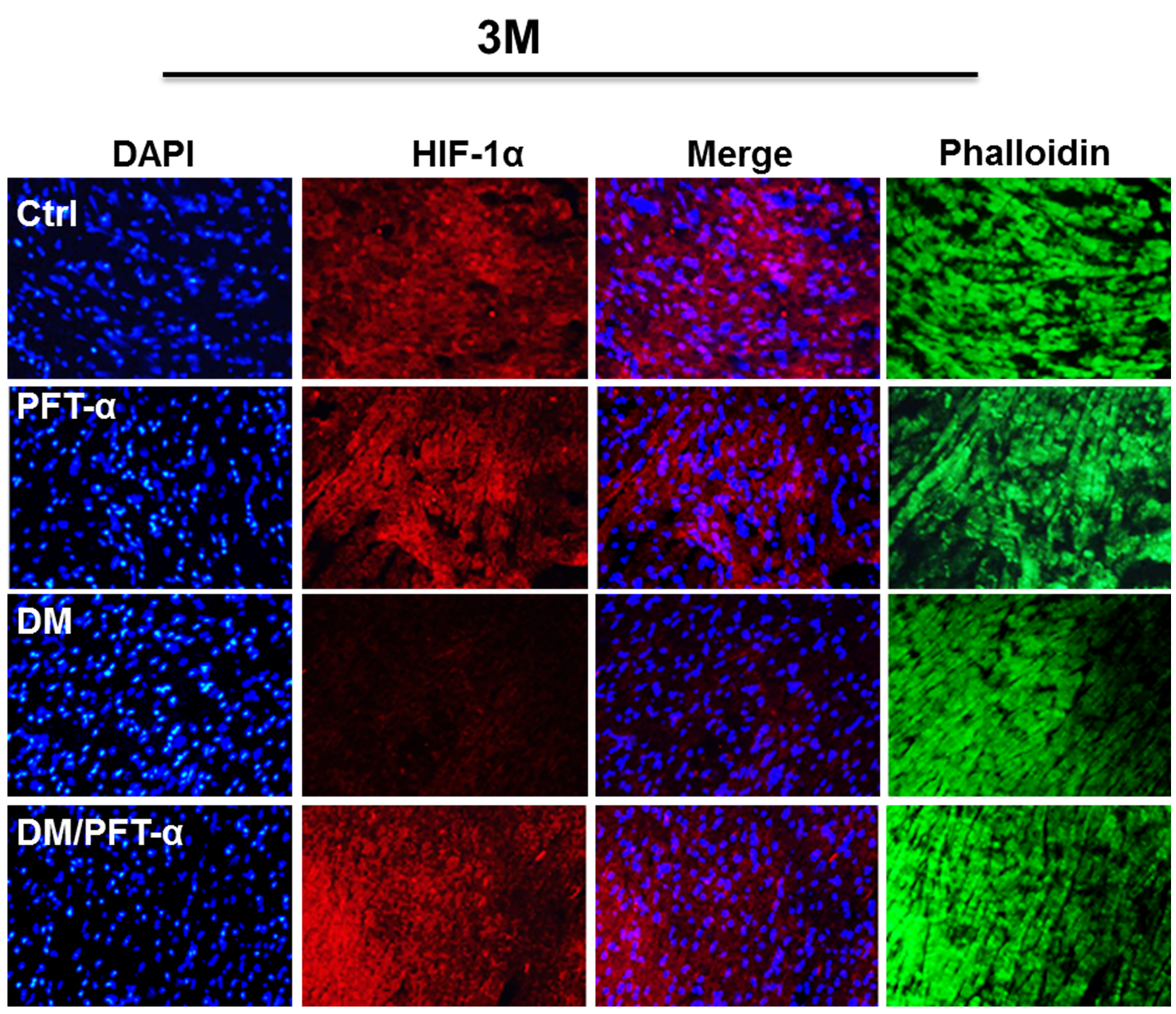


Supplemental Fig. 5. Effect of PFT-α treatment on HIF-1α protein expression at 3 month after diabetes onset.


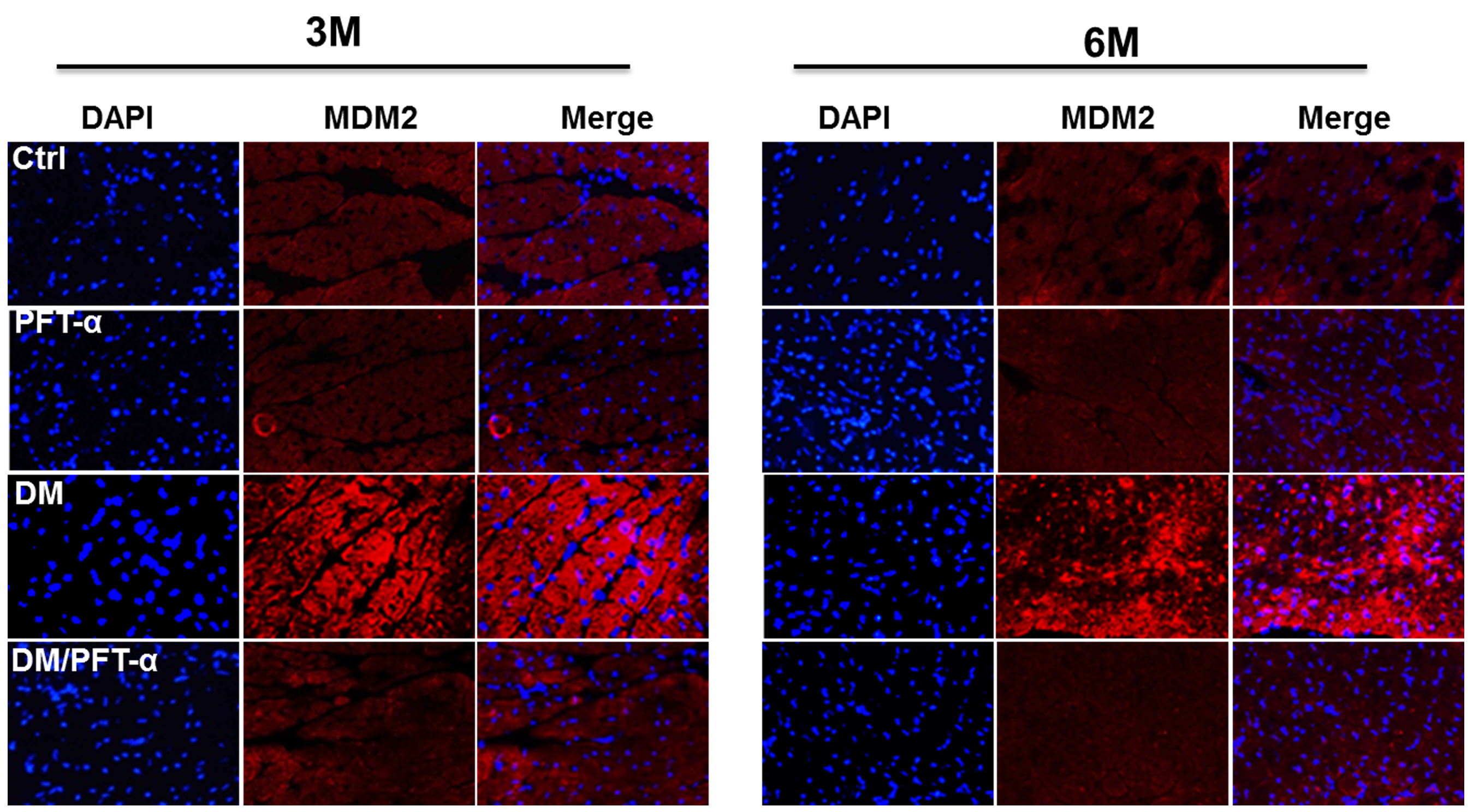


Supplemental Fig. 6. Effect of PFT-α treatment on MDM2 protein expression at 3- and 6-month after diabetes onset


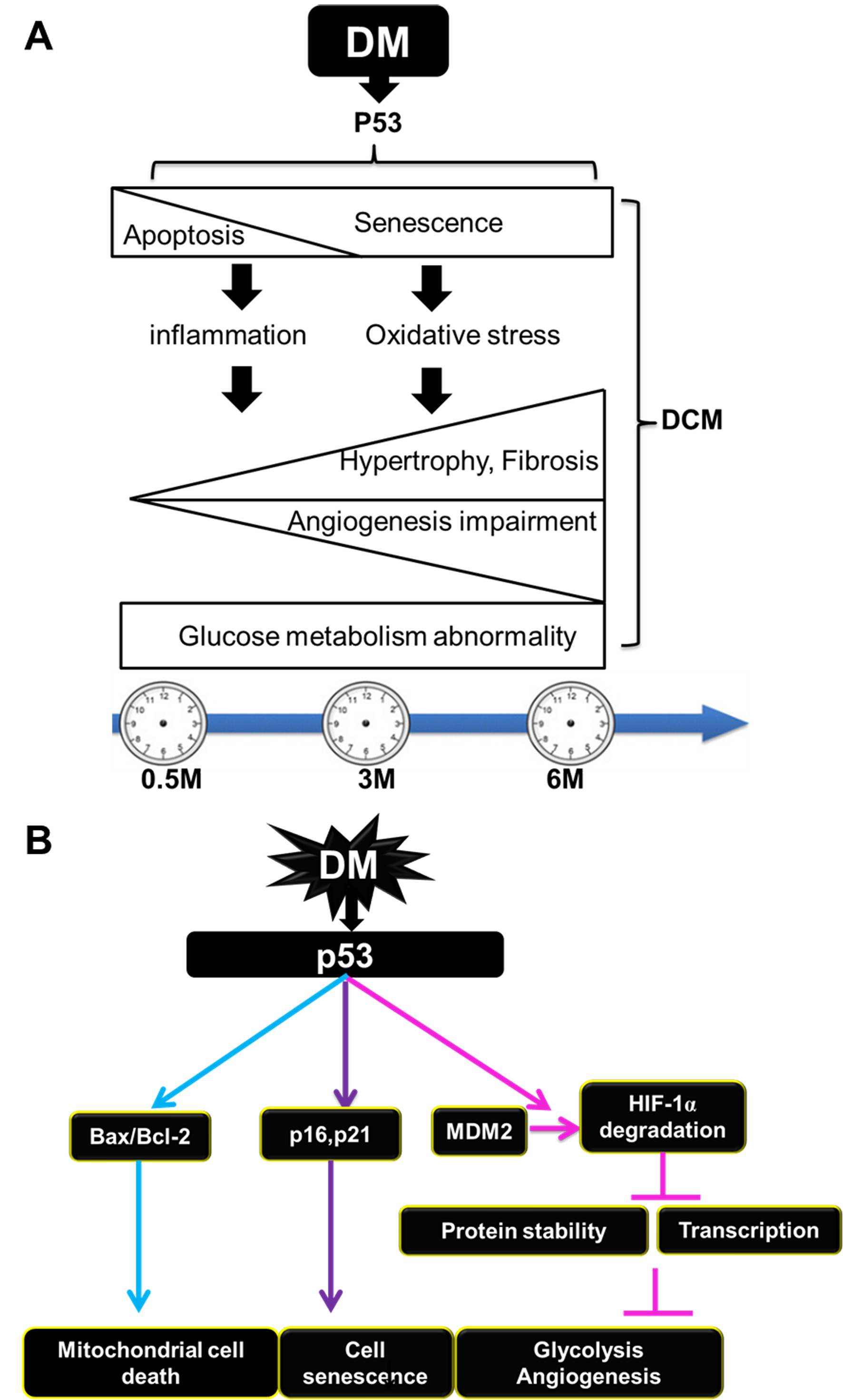


Supplemental Fig. 7. Schematic illustration for the cardioprotective effects (A) by p53 inhibition and the underlying mechanisms (B) by which p53 inhibition prevents the development of DCM.
